# ART implantation failure and miscarriage in patients with elevated intracellular cytokine ratios: response to immune support therapy

**DOI:** 10.1186/s40738-018-0052-6

**Published:** 2018-10-17

**Authors:** Conor Harrity, Lyuda Shkrobot, David Walsh, Kevin Marron

**Affiliations:** 1 Royal College of Surgeons Ireland, 123 St Stephen’s Green, Dublin 2, Ireland; 2 Sims IVF Clinic, Clonskeagh Road, Clonskeagh, Dublin 14, Ireland

**Keywords:** Immunomodulation, Cytokines, Implantation, Miscarriage

## Abstract

**Background:**

The origins of adverse reproductive outcome can be multifactorial, but the contribution of the maternal immune system is considered debatable. Elevated intracellular cytokine ratios have been proposed, although not universally supported, as a marker for immunological dysfunction in implantation and early pregnancy. Poor patient selection or inadequate treatment or testing may be confounding factors. Specific immunomodulation, in carefully selected sub-populations of ART patients with poor reproductive history, despite transfer of good quality blastocysts, may potentially improve clinical outcomes.

**Methods:**

Intracellular cytokine ratios (CKR) were prospectively assessed in 337 patients presenting with a history of implantation failure and/or pregnancy loss, prior to further treatment, and were found to be elevated in 150 (44.5%). Of this group, 134 agreed to initiate a standardised immunotherapy regime (nutraceuticals, prednisolone & intralipids) to evaluate the efficacy of this proposed therapy. Of the intervention population, a small cohort (*n* = 70) delayed commencing ART for ~ 10 weeks to assess if extended pre-treatment nutraceutical supplementation could normalise CKRs prior to starting ART, and if this conferred additional benefit.

**Results:**

Baseline assessment in the intervention population (*n* = 134) identified 160 miscarriages from 180 total pregnancies (89% miscarriage rate, MR), conceived both spontaneously and by assisted reproduction. Post-treatment analysis of subsequent ART cycles revealed a significant improvement in both implantation (OR 3.0, 2.0–4.5) and miscarriage rates (41/97, 42.2% MR, *P* < 0.001). Interestingly, pre-treatment normalisation of CKRs appeared to impart marginal extra benefit prior to subsequent fertility treatment with immunotherapy.

**Conclusions:**

Following immunomodulation, significant improvements in both implantation rate and miscarriage rate were seen in this poor prognosis population. This suggests a possible role for both detailed immuno-evaluation of patients with poor reproductive history with good embryo quality, and application of personalised immunotherapy regimes alongside ART in selected cases. Future randomised controlled trials are needed to definitively evaluate this potentially promising therapeutic approach.

## Background

Maternal immunological modulation in pregnancy is a well-established concept [1–4], but interpretation of variations in the immune status of those attempting to conceive but appearing to have implantation failure of good quality embryos, or with recurrent pregnancy loss, is controversial [5–7] Many factors are accepted as necessary for the successful completion of an assisted reproductive technology (ART) cycle from implantation to birth. Oocyte and sperm quality along with the endometrial environment are a few examples of the hurdles which must be cleared early in the process, while maternal allograft suppression and thrombotic risk must be achieved later [8]. The active and particularly innate immune systems are accepted components of this process and many studies link various immunological abnormalities with recurrent implantation failure (RIF) and recurrent pregnancy loss (RPL) [9–12]. Auto-immune factors such as anti-phospholipid antibody syndrome and anti-thyroid peroxidase antibodies have also been shown to influence pregnancy outcome [13–16] and empirical treatment in these cases is recommended [17]. Although not standard practice, testing for immunological abnormalities in the lymphocyte populations specifically, is gaining more attention in this field. Circulating T cells, cytokine expression bias and natural killer cells are known to be fundamental to a successful pregnancy [18] so in this regard, testing of this nature seems justified. NK cytotoxicity, for example, has been reported as being predictive of RPL [19]. Glucocorticoid steroids have long been the agents of choice in attempts to influence the expression and activity of pro-inflammatory mediators and have been used as a key component of many proposed immunomodulatory regimens [15, 20–22]. Similarly, IVIG and intralipid infusions have been used as adjunctive agents to both supress and confuse the maternal adaptive immune system around the window of implantation [23]. Intralipid has been proposed to exert a modulating effect on certain immune cellular mechanisms, potentially down-regulating cytotoxic or activated natural killer cells (NKa) [24]. This effect may act synergistically with the concomitant administration of corticosteroids, such as dexamethasone or prednisolone, to suppress cytotoxic/activated T-lymphocytes. A potential net effect of combined intralipid and corticosteroid therapy might be the ability to suppress pro-inflammatory cellular (Type-1) cytokines such as interferon gamma and TNF-alpha. Indeed, in-vitro testing has shown that Intravenous Intralipid administration can successfully downregulate natural killer cell activation (NKa) within 2–3 weeks in up to 78% of women experiencing suspected immunologic implantation dysfunction [25–27].

The capacity to produce monomeric cytokines, either pro-inflammatory, (TNF-α, IFN-γ) or anti-inflammatory (IL-10), exists in many lymphocyte subsets [28]. Stimulated intracellular cytokine expression levels in peripheral blood lymphocytes, specifically CD4+ T cells, has shown an association between the levels of expression of these markers and the incidence of both recurrent spontaneous abortions and recurrent implantation failure [29]. Intracellular cytokines of this nature are known to stimulate activatory KIR receptors on NK cells and thus render them more cytotoxic [30]. Pharmaceuticals such as Humira® (adalimumab) and Enbrel® (etanercept) are sometimes used in the miscarriage/implantation failure patient who presents with raised intracellular cytokines [31], but not without controversy due to their toxicity profile and the lack of general acceptance for requirement of this course of action. The immunomodulatory regime employed in this study is an attempt to provide a similar physiological response, an improved outcome and a reduced risk of adverse effects.

There is some evidence that complementary treatment with appropriate supplements and anti-oxidants, such as Vitamin D3, can also improve the natural conception rate of sub-fertile couples, and increase the success rate of assisted reproductive techniques [32]. Vitamin D is a well-established immune modulator and may have an influence on reproductive capacity, with deficiency associated with autoimmune problems [33] and recurrent pregnancy loss (RPL) [34]. Vitamin D has many functions but in the reproductive setting it is thought to regulate T helper cells and decrease Th1 response while promoting suppressor Th2 cells, thus helping the body maintain a pregnancy [35]. Omega 3 fish oils, especially Eicosapentaenoic acid (EPA), have been shown to potentially decrease NK cell activity by up to 48% [36]. It may also inhibit in vivo TNFa by up to 74% [37], with cytokine production decreasing as EPA levels rise. The mediators of all these effects are thought to be modifications to the production and release of pro-inflammatory cytokines such as TNFa and IFNg, which are primarily the purview of CD4+ T cells and NK cells. These studies led us to hypothesise that treatment with glucocorticoids and intralipid infusions, combined with Vitamin D3, and omega 3 fatty acids in conjunction with B complex vitamins may have a positive immunomodulatory effect, and benefit some patients in achieving and maintaining pregnancy. By focusing our observations on selected RPL/RIF subpopulations, where potential immune derangement is more likely, especially with baseline cytokine ratio elevation, specific and appropriate patient subgroups may be identified where immunomodulation can be applied to best effect.

## Methods

The study design was a longitudinal prospective cohort comprising a full historical evaluation of patient demographics and outcomes at presentation, followed by intracellular cytokine testing for inclusion, if appropriate, and subsequent treatment with additional immunotherapy in those with abnormal baseline levels. Patients presenting with a history of early pregnancy losses or failed embryo transfers were offered an initial assessment of their CD4+ intracellular cytokine ratios (CKR) prior to their next ART cycle using the investigative technique as previously described [38]. Enrolment took place in a single private university affiliated ART centre over a 3 year period, from March 2013 to March 2016. Inclusion criteria for offering this test was a background of either > 2 miscarriages or minimum 2 unsuccessful transfers of good quality embryos, with female age < 42 years. An anatomically normal endometrial cavity on saline infusion sonogram and normal endocrine profile (thyroid function and prolactin within normal ranges) were also required. Normal ranges for interpreting CKR’s were based on levels established originally by Rosalind Franklin Labs, Chicago, and are reported as 13.2–30.6 for TNF-α: IL-10 and 5.8–20.5 for IFN-γ: IL-10 [29]. Levels above these established ratios are considered “High”. We also report the individual percentage expression levels which constitute the ratios. Freshly collected whole blood cells were diluted into Iscoves modified Dulbecco’s medium (IMDM, Gibco Life technologies) fortified with 5000 IU penicillin & streptomycin (Gibco life technologies) and separated into stimulated and un-stimulated groups. The stimulated aliquot had 50 ng/ml phorbol myristate acetate (PMA) and 1 micromol/L ionomycin added in the presence of 1 uL Golgi Plug protein transport inhibitor (Sigma Aldrich, UK) to allow for protein accumulation in the Golgi complex. The unstimulated group had only the Golgi Plug introduced. Incubation took place overnight for ~ 18 h at 37° centigrade in 5% CO2. All cells were then processed and stained according to the manufacturer’s instruction with the BD whole blood Cytotoxic/Cytoperm kit (BD Pharmingen). Analysis on the Navios™ 10 colour 3 laser flow cytometer (Beckman Coulter UK) then followed immediately using dedicated software. Post incubation, the cells were lysed in Pharmlyse buffer and following washing and centrifugation steps, were incubated with antibodies to surface ligands CD45 (anti-human Krome Orange, clone J.33), CD3 (anti-human Pacific Blue clone UCHT1) and CD8 (anti-human PC7 Clone B9.11), followed by a fixation and permeabilisation step. To detect intracellular cytokines, monoclonal antibodies to TNF-α, IFN-γ and IL-10 were employed, specifically phycoerythrin (PE)-anti-human TNF-α clone IPM2, Fluorescein isothiocynate (FITC)- anti- human IFN-γ clone 45.15 (both Beckman Coulter UK) and allophycocyanin (APC)-anti human IL-10 clone JES3-19F1 (BD Pharmingen). Corresponding isotype controls were utilized for each antibody and for each patient as well as fluorescence minus one (FMO) controls.

Included patients were stratified into subgroups. Recurrent pregnancy loss was defined based recent on ESHRE guidelines of 2 or more miscarriages prior to 22 weeks [39]. Ultrasonic evidence, in addition to positive hCG, denoted clinical pregnancy [40]. As the RPL population is a heterogeneous group encompassing many aetiologies, we proposed a further subdivision into patients with non-consecutive miscarriages (RM), two consecutive miscarriages (RM2) and those with more (RM3+) to determine if differences were present. Implantation failure (IF) and repeated implantation failure (RIF) are concepts unique to assisted reproduction, with many factors important in the assignment of this terminology, including early bhCG evaluation, number of embryos transferred and number of previous IVF failures [41]. RIF for the purposes of this study was categorised as a background of primary infertility, combined with the published definition of a minimum of two unsuccessful blastocyst transfers following at least two attempts [42]. Despite these entry criteria there does exist a third population which cannot be clearly assigned to either the RPL or RIF group and these have been denoted as “Other”. These patients presented with secondary infertility and a either a mixed combination of IF and RPL, or new onset implantation failure after previous live birth.

**Fig. 1:**
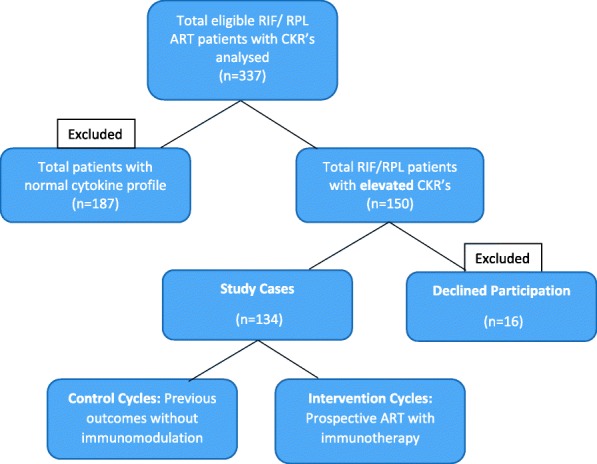
Flow chart representing patient enrolment. Only those patients who met the entry criteria AND presented with elevated CKR’s were enrolled in the study. The entry criteria for CKR assessment were a history of RIF and/or RPL. All participants received standard ART techniques depending on age, AMH, BMI etc. ART; assisted reproductive techniques, RIF; repeat implantation failure, RPL; recurrent pregnancy loss

A total of 337 patients were recruited for cytokine analysis, of which 44.5% (*n* = 150) had ratios above the described normal range, and so were eligible for inclusion in the study (Fig. 1). Enrolment was confirmed after detailed discussion of the potential risks and benefits of using the interventional therapy, with 16 patients declining to take part. The treatment arm was commenced on an immunomodulatory regime of prednisolone (15–25 mg started with ovarian stimulation or estrogenic endometrial preparation), continuing until 12 weeks of gestation or negative test. Intravenous intralipid infusions (Fresenius Kabi, 20% *w*/*v*, administered biweekly-1 week pre- and 1 week post-embryo transfer, continuing with a final third dose after confirmation of pregnancy). An immune influencing nutritional regimen (Omega 3 3 g, B complex, Vitamin D3 25μg/1000iu, daily) was used in a subset of patients willing to delay empiric therapy for approximately 10 weeks. Responses to this approach are previously described [38]. Addition of low molecular weight heparin (Enoxaparin/Clexane 20–40 mg SC as per BMI) was utilised, if appropriate, based on thromboelastogram result (INTEM-s ® MCF result ≥64 mm) [43]. Controlled ovarian stimulation was performed using long agonist (Buserilin™) or short antagonist (Cetrotide Merck) protocols, with either recombinant FSH (Gonal-F™, Merck UK Ltd) or hMCG (Menopur™, Ferring LTD) based on age, AMH, baseline gonadotropin levels and antral follicles count. Final oocyte maturation trigger (Pregnyl™, MSD or Oviterelle™, Merck UK LTD) was administered once ≥2 follicles were ≥ 17 mm, and progestogenic luteal phase support was added (Crinone™/ Gestone™) and continued until negative pregnancy test, or 12 weeks gestation. Frozen embryo transfer cycles were performed using a HRT protocol without pituitary downregulation with transfer at P + 5. All donor oocyte treatments were performed following this approach. Clinic practice was to recommend routine extended embryo culture to blastocyst for transfer. PGT-A was not available in the centre during the study period, so not performed on either control or interventional cycles.

A small group of patients (*n* = 16), declined to take part in the study after full counselling despite having elevated cytokine ratios, and proceeded directly to ART as per standard unit protocols, without the addition of immunosuppressive therapy. Control cycles were defined as those ART cycles occurring historically prior to initial CKR analysis where no immunotherapy was used, with both internal clinic data and external centres included, along with spontaneously conceived pregnancies. The interventional arm comprised the subsequent cycles in the same patients, following the CKR evaluation and using the empirical immunotherapy regime described above. Prospective observation was performed on the interventional cycles, with analysis of subsequent pregnancy and miscarriage rates (Table 1). Informed consent was obtained from patients for the analysis and study approval was obtained from the Clinic’s Institutional review board.

**Table 1:** Baseline demographics

Patient demographics	Treatment Arm	Excluded Cases
Age (Mean ± SD)	37.8 ± 4.7	37.7 ± 6.1
Total patients (n)	134	16
Classification (n(%))		
Repeat Implantation failure (RIF)	56 (41.0)	6 (37.5)
Recurrent Pregnancy Loss (RPL)	46 (35.1)	6 (37.5)
Other	32 (23.9)	4 (25.0)
Pre-intervention obstetric history		
Total pregnancies	180	3
Miscarriages	160	21
Miscarriage rate	*88.9%*	*87.5%*
Post-intervention outcomes		
Total pregnancies	103	2
Miscarriages	44	2
Miscarriage rate	*42.7%*	*100%*

Baseline demographics in patients with elevated CKR’s (*n* = 150). Patients treated with immunotherapy in addition to standard ART comprise the study population (*n* = 134) while those who declined immunomodulation (*n* = 16) were excluded

Statistical analysis was performed using SPSS v 24.0 to identify if any significant differences were noted. Cytokine data was determined to not be normally distributed so non-parametric analysis by Kruskal Wallis testing was utilised to compare levels between patient subgroups. Chi square analysis was used to compare proportions, with McNemar’s test applied as a repeated measures method to account for multiple pregnancies for individual patients when appropriate. Odds ratios were used to assess the strengths of associations. As there is no central birth register for ART pregnancies in Ireland, ongoing pregnancy at 12 weeks’ gestation was chosen to be the primary outcome measure. Patients were referred on to regional maternity units after this stage making follow up more difficult. The majority of miscarriages, however, are expected to occur in the first trimester, with later losses considered less likely.

## Results

During the study time period 337 eligible patients had CKRs assessed, and were found to be elevated in 150 (44**.**5%). After medical counselling 134/150 elected to use the study therapeutic regime, 16 declined the addition of immunomodulation and study participation and so were excluded from the analysis. PGT-A was not utilised in any cases. There were no significant differences in demographics between patients joining the intervention group and the excluded cases, so no bias was introduced when these patients elected not to procced in the study (Table 1). None of the included patients tested true positive for antiphospholipid antibodies, specifically anti-cardiolipin IgG and anti b2 glycoprotein 1. There were no Mullerian tract abnormalities found on initial endometrial cavity assessment. Potential coagulation disorders were assessed using maximal clot formation (MCF) via thromboelastogram (ROTEM™, Werfen UK LTD), with the threshold for miscarriage risk as described by Rai et al. [43], and treatment with low molecular weight heparin (Enoxaparin/Clexane 20-40 mg as per BMI) added if appropriate. The intervention group consisted of 47 (35%) patients with a classical history of recurrent pregnancy loss, while 55 (41%) met the criteria for repeated implantation failure. Thirty-two patients (24%) met the overall study inclusion criteria but could not be clearly classified into either RPL or RIF groups and are deemed “other”. Baseline assessment of the studied population (*n* = 134) showed elevated CKRs across the treated groups irrespective of clinical presentation (Table 2). There was no difference in TNFa:IL10 or IFNg:IL10 ratios for the RIF and RPL groups, however RPL patients demonstrated significantly higher percentage expression of TNFa (*P* = 0.036). Regarding coagulation status there was also no significant difference, with the RPL group having a mean MCF of 65.2 (range 62–75), compared to 64.7 (range 62–70) for the RIF patients.

**Table 2:** CKR expression across patient aetiology

Group	Cytokine expression: M ($\overline{\mathrm{x}}$)				
	TNFa:IL10	IFNg:IL10	IL-10	TNFa	IFNg
	(ratio)	(ratio)	(%)	(%)	(%)
RIF	63.6 (54.0)	16.7 (21.8)	0.80 (0.87)	44.9 (49.8)	15.3 (17.3)
RPL	54.8 (62.6)	20.1 (23.0)	0.95 (0.93)	55.2 (53.2)	18.2 (21.2)
Other	68.4 (72.0)	21.8 (23.4)	0.60 (0.71)	42.2 (48.4)	13.7 (15.6)
*Sig. (P)*	*0.086*	*0.470*	0.027*	0.036*	*0.315*

Mean baseline intracellular cytokine ratios and percentage expression in the study group (*n* = 134), stratified by patient aetiology. ANOVA demonstrates no significant difference in baseline stimulated intracellular cytokine expression or ratios between the populations. Only patients with elevated ratios met study inclusion criteria. RIF patients presented with repeated implantation failure (no pregnancy following at least 2 transfers of good quality embryos). RPL are patients with recurrent pregnancy loss. “Other” refers to secondary infertility patients with features of RIF/RPL, not meeting the strict criteria for either group. *indicates statistical significance to *P* < 0.05

Prior to immunotherapy initiation, historical assessment of the intervention group (*n* = 134) identified 160 previous miscarriages from 180 total pregnancies, and an extremely high miscarriage: pregnancy ratio (MR) of 89% (Table 1). Subsequent analysis of the post-treatment cycle outcomes in the treated population revealed that 103 pregnancies were achieved, with 44 ending in miscarriage, resulting in a MR of 42.7% (*P* < 0.001, Table 3). There was no difference in MR between the RIF and RPL groups after treatment (*p* = 0.97). When the RPL group was subdivided, there was no difference in either outcomes or baseline cytokine levels between those with 2 consecutive miscarriages or those ≥3. Due to the very small group sample size (*N* = 2), it was not possible to conclude if those with non-consecutive miscarriages form a distinct population or not.

**Table 3:** Pre- and post-treatment outcomes stratified by patient aetiology

	n	Control Outcomes (Pre)		Intervention Outcomes (Post)		Sig (P)
		Pregnancies	Miscarriages (MR%)	Pregnancies	Miscarriages (MR%)	
RIF	56	0	0 (NA)	44	16 (36.4)	0.015
RPL	46	148	137 (92.6)	33	13 (39.4)	*< 0.0001*
*- RM2*	*25*	55	51 (92.7)	17	6 (35.3)	*< 0.0001*
*- RM3+*	*19*	*87*	*82 (94.3)*	*15*	*7 (46.7)*	*< 0.0001*
Other	32	32	23 (71.9)	26	15 (57.7)	0.50
Total	134	180	160 (89.0)	103	44 (42.7)	*< 0.0001*

Population outcomes in the study population (n = 134) both before (Control outcomes) and after (intervention outcomes) immunomodulation, stratified by patient aetiology. RIF patients presented with repeated implantation failure (no pregnancy following at least 2 transfers of good quality embryos). RPL are patients with recurrent pregnancy loss, the RM2 and RM3+ subgroups refer to either 2 or ≥ 3 consecutive losses, while the remainder are non-consecutive losses. “Other” refers to secondary infertility patients with features of RIF/RPL, not meeting the strict criteria for either group. Significance calculated using McNemar’s test

Interestingly, implantation rates for the entire cohort demonstrated significant improvements, with an Odds Ratio of 3.0 (95% CI 2.0–4.5) for successful implantation following immunotherapy treatment in those with high baseline cytokine ratios. When stratified by ART type, the differences were most prominent with FET and donor oocyte transfer cycles following immunomodulation (Table 4). Of note, the multiple gestation rate increased despite a reduction in the numbers of embryos being transferred. Pre-treatment the average number of embryos transferred was 1.66, with a twin incidence of 4%. Post immunomodulation the average number of embryos per transfer was lower, reducing to1.38, yet with a higher twin pregnancy rate of 14.7%.

**Table 4:** Pre- and post-implantation outcomes stratified by treatment modality employed

	Control Cycles (Pre)				Intervention Cycles (Post)				OR (95 %CI)	Sig (p)
	ETs	Embryos	GSs	IR (%)	ETs	Embryos	GSs	IR (%)		
IVF	75	125	25	20.0	55	79	22	27.8	1.54 (0.8–3.0)	0.196
ICSI	48	87	9	10.3	69	80	15	18.8	2.0 (0.8–4.9)	0.127
FET	30	44	1	2.3	67	96	28	29.2	*17.7 (2.3–134.9)*	*0.006*
DE	24	39	3	7.7	60	92	41	44.6	9.6 (2.8–33.6)	*0.0004*
Overall	*177*	*295*	*38*	*12.9*	*251*	*347*	*106*	*30.5*	*3.0 (2.0–4.5)*	*0.0001*

Implantation rates (IR) in the studied group of 134 patients, before (Pre) and following (Post) immunomodulatory therapy. Significant improvements seen in IR following immunomodulatory therapy in FET and particularly DE cycles. *ETs* Embryo transfers, *GSs* Gestational sacs seen, *IVF* in-vitro fertilisation cycles, *ICSI* intracytoplasmic sperm injection cycles, *FET* Frozen embryo transfer cycles, *DE* Donor egg cycles

**Fig. 2:**
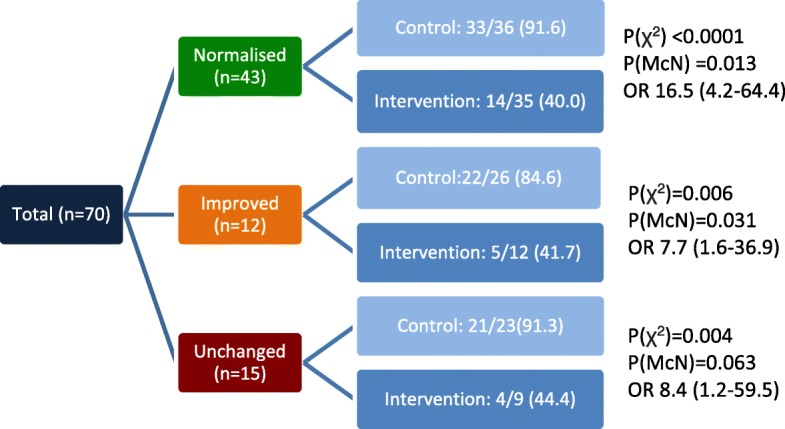
Cytokine changes and pregnancy outcomes in the patient subset who received nutraceuticals. A cohort of patients (70/134) received a 10 week nutraceutical regime consisting of high dose omega 3, Vitamin D and Vitamin B6 followed by repeated cytokine analysis prior to ART with immunomodulatory intervention (prednisolone and intralipid infusions). This chart illustrates the breakdown of pre- treatment (control) and post- treatment (intervention) pregnancy outcomes for patients who achieved normalisation, improvement or no change in their CKR levels. Significance levels calculated using Chi Square (χ^2^), McNemar’s test (McN), and Odds Ratio (OR) for reduction in miscarriage following immunomodulation

A subgroup of 70/134 patients from the intervention group elected to attempt to normalise their CKR levels with extended nutraceutical supplementation therapy prior to commencing pharmacological immunomodulation and ART, as previously described [38]. At baseline assessment 39 of these patients showed raised TNFα and IFNγ ratios (high/high) and 31 showed raised TNFα but normal IFNγ  ratios (high/norm). Following 10 weeks of pre-treatment, considerable normalisation was achieved, with 43/70 reaching normal or normal/low levels, 12 being partially responsive, and 15 remaining unchanged. Nutraceutical pre-treatment led to an average 6 fold increase in IL-10 expression (5.0% vs 0.8%) and a decline in absolute percentage expression of 10.6% for TNFα and 4.6% for IFNγ ([38]). Interestingly, this subset analysis showed that cytokine ratio normalisation conferred only marginal additional benefit to the change in miscarriage rate following immunomodulation (Fig. 2). All groups showed a > 50% reduction in miscarriage: pregnancy ratio after standard immunosuppressive therapy, regardless of their change in CKR. Chi square testing showed this was significant in all categories, however when McNemar’s test was applied the reduction in MR in patients with unchanged cytokine levels (91.3% to 44.4%) was only approaching statistical significance (*P* = 0.063). Odds ratios, however, demonstrate the highest level of improvement in patient’s whose cytokine ratios normalised (Table 4).

## Discussion

Reproductive immunology is on the fringe of accepted medical practice in the world of assisted reproduction, and for every advocate there are many naysayers [5]. The presence of a dominant Th1 type phenotype in patients with recurrent miscarriage or implantation failure is a proposed common underlying pathology [29, 30]. Controversy exists, however, as to the generality of testing in peripheral blood and its relevance to the uterine environment [6, 44, 45]. There are many methods available looking at varied markers in both peripheral blood and endometrium, assessing both percentage expression and cell numbers of lymphocytes [46]. Stimulated intracellular cytokine ratios in peripheral blood, however, remains a frequently performed test, but is controversial with many experts not advocating its use. We have previously shown that a dietary regime can be beneficial in significantly reducing intracellular cytokine ratios. Similar TNFa:IL-10 modifications with positive outcomes have been described in other populations [47]. Surprisingly, the movement of intracellular cytokines towards a normal level is not the only influential factor in improving reproductive outcomes, as even the subgroup whose ratios remained unchanged showed a large and significant reduction in miscarriage rates. Rather, it appears that the regime of prednisolone, intralipid infusions and LMWH (if indicated), could be the key influencers. Given the quick transition to a full immunomodulatory regimen, however, it is possible that the effect of the dietary pre-treatment may not yet be fully realised, and a longer preparatory phase may be useful. A broad spectrum immunotherapy regime was devised to tackle potential issues via several possible pathways and mechanisms of action. The aim of this was to maximise the therapeutic benefit of our immune modulatory protocol, however, a detrimental limitation is that it becomes difficult to assess the relevant contribution of each agent and to determine which individual therapy has the most beneficial influence. Further research is required to determine which agents, either alone or together, could potentially have the greatest effect. The ability to determine the relative effect of each component would allow a more personalised protocol for each individual patient. Studies assessing the individual relative benefits of the empiric treatments described here have previously been conducted with variable findings both advocating and not advocating their use [21, 48, 49]. The success of the regimen employed here as evidenced by improved implantation, successful pregnancies and a dramatic reduction in the overall miscarriage rate demonstrates both the combined beneficial effect of these agents as well as the potential importance of specific immunological testing in selected cases. The origin of the underlying difficulty in these patients, be it implantation failure, early versus late miscarriage or consecutive miscarriages, may be very different. Peripheral blood immunophenotyping to an even deeper level, perhaps encompassing KIR receptors, is becoming more achievable, however, it is possible that endometrial biopsy evaluations may be even more helpful in the evaluation and treatment of these difficult cases. The use of TNFa inhibitors such as adalimumab (Humira®, AbbVie) may be of use in the populations that either do not respond to, or continue to have an adverse outcomes with this standard regime, but certainly further research on this approach is also needed. A prospective, blinded, randomised controlled trial, allocating patients with elevated CKRs to the standardised immunomodulation/ nutraceutical regime, or a placebo, would be the way to definitively confirm the findings of this study.

The data demonstrates that in this high-risk population, a significantly higher implantation and pregnancy rate is achieved in patients empirically prescribed a standardised immunotherapy regime compared to the same patients who previously had similar ART procedures without additional immune supports approximately 2 years previously. A delay of this magnitude would generally be accepted as affording a poorer prognosis to these patients rather than an improved one. The success of the regime in this population may, in part, be ascribed to more discerning immunophenotyping.

## Conclusion

Elevated cytokine ratios have been linked with adverse reproductive outcomes. Proposed treatment protocols have often utilised biological immunomodulators, which antagonise TNFa, but these agents have cost implications, and potentially cytotoxic side effects, particularly with repeated or prolonged exposure. A combination regime with a lower associated risk profile could be more patient friendly, safer, less controversial, and have better compliance. A standardised immunotherapy regime may lead to improved obstetric outcome for a proportion of patients identified to be high risk at baseline testing, reserving the need for more aggressive treatments to those who do not respond to first line interventions, such as those with high order RPL. Unfortunately, the study data is limited by small sample size and lack of randomisation, but the findings are very promising and would suggest that a large prospective randomised controlled trial, to eliminate potential bias and confounding factors, would be useful to determine if this theory is indeed credible. We feel we have identified a population subgroup in which our approach is merited, and with appropriate and timely interventions can allow these patients to realise their strong desire to become parents.

## Abbreviations

ART: Assisted reproductive technologies
CKR: Intracellular cytokine ratios
EPA: Eicosapentaenoic acid
IF: Implantation failure
IFNg: Interferon gamma
IL-10: Interleukin 10
IVF: in-vitro fertilisation
LMWH: Low molecular weight heparin
MR: Miscarriage: Pregnancy Ratio
NK cells: Natural killer cells
RIF: Repeat implantation failure
RM: Recurrent Miscarriage (non-consecutive)
RPL: Recurrent pregnancy loss
T cells: T lymphocytes
Th1: T helper 1
Th2: T helper 2
TNFa: Tissue necrosis factor alpha
